# Undesired effect of excessive betamethasone administration during pregnancy: A rare case 

**Published:** 2018-03

**Authors:** Alireza Mirzaei, Solmaz Piri, Kaveh Gharanizadeh, Mozhdeh Zabihiyeganeh

**Affiliations:** 1 *Bone and Joint Reconstruction Research Center, Shafa Orthopedic Hospital, Iran University of Medical Sciences, Tehran, Iran.*; 2 *National Association of Iranian Gynecologists and Obstetrician, Tehran, Iran.*

**Keywords:** Osteoporosis, Postpartum, Femoral neck fracture, Twin, Pregnancy

## Abstract

**Background:**

Postpartum bilateral femoral neck fracture (BFNF) is a rare condition. We here report a case of BFNF due to excessive corticosteroid consumption, twin pregnancy, immobility, and vitamin D deficiency.

**Case:**

This is a report of a 32-yr-old woman with bilateral femoral insufficiency fracture five days after emergency cesarean section due to preterm labor, twin pregnancy, and the history of a previous cesarean section at 33 wk. Antenatal repeated courses of betamethasone injections for fetal lung maturity, daily oral use of prednisolone for the history of miscarriage, immobilization, and vitamin D deficiency were the important contributing factors in her past medical history and lab investigations. The bone mineral density examination showed low bone density for the expected age.

**Conclusion:**

Clinicians, who deal with pregnant women, should consider the diagnosis of bilateral femoral insufficiency fracture in any pregnant women with pelvic pain. Awareness of risk factors of BFNF might help to reduce the rate of this complication.

## Introduction

Simultaneous bilateral femoral neck fracture (BFNF) is an uncommon condition which has been reported to be caused by generalized epilepsy, osteomalacia, hypovitaminosis D, and chronic renal failure. According to the earlier reports, the majority of BFNF cases in postpartum period were the consequence of transient osteoporosis of pregnancy (1, 2). 

During the third trimester of pregnancy and lactation period, the considerable fetal consumption of calcium and vitamin D will be met mostly through re-absorption from the maternal skeleton. In this condition, the drastic loss of bone mass suddenly presents, which can lead to the osteoporotic fractures during pregnancy or thereafter (3). 

We report a case of bilateral femoral neck insufficiency fracture in a 32-yr-old woman following a cesarean section. To our knowledge, there was no reported case of BFNF associated with pregnancy in Iranian pregnant women.

## Case report

**Figure 1:**
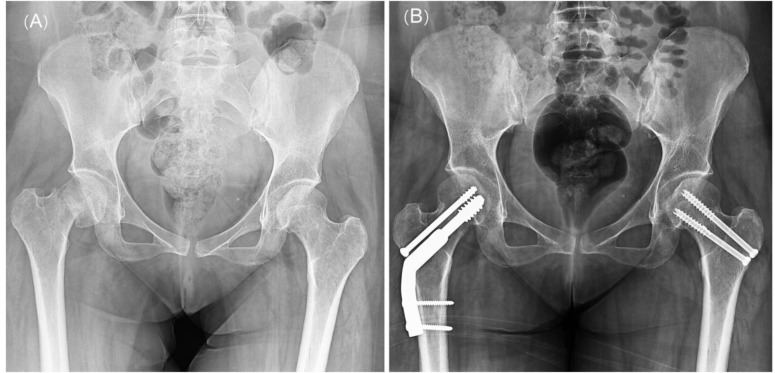
(A) Pre-operative radiograph of the femur showing right femoral neck fracture. Left femoral neck fracture line was only identifiable in the MRI; (B) Post-operative radiograph of the femur, showing the management of right femoral neck fracture with CRIF and DHS and the management of left femoral neck fracture with CRIF and screw.

A 32-yr-old woman was referred to our center with the inability of weight bearing, five days after primary cesarean section for her twins. She had recently given birth to a pair of healthy twins at 33 wk of pregnancy and had two other pregnancies leading to another healthy child and a miscarriage. Her body mass index was 25 kg/m^2^. Physical examination showed the severe limitation of motion in the right hip and moderately painful left hip. Right femoral neck fracture was clearly identified in the X-rays. In pelvic magnetic resonance imaging, fracture lines at both femoral necks along with peripheral bone edema were visualized, confirming the presence of a simultaneous fracture in the left femoral neck of the patient too. The fracture of the right femoral neck was managed by close reduction and internal fixation (CRIF) along with dynamic hip screw, while CRIF and screw were used for the fixation of left femoral neck fracture (Figure 1). 

Since no history of trauma was reported by the patient, she was referred to the rheumatologist, where further investigations of the underlying cause of the fractures were performed. The positive past medical history points were repeated weekly courses of antenatal Betamethasone injections for fetal lung maturity, started from 26 wk gestation, plus daily oral 5 mg prednisolone due to the history of a previous miscarriage and complete bed rest during the whole third trimester of pregnancy.

In the laboratory evaluation, patient serum calcium, phosphorus, creatinine, alkaline phosphatase, parathyroid hormone and 24 hr urine calcium were normal; Serum vitamin D was 15 ng/ml, which was compatible by vitamin D deficiency. The results of bone mineral density showed a Z-score of -2.2 in L1-L4, suggesting low bone mineral density (Z score <-2). The woman was advised to discontinue breastfeeding, while her medical treatment was initiated with a daily subcutaneous injection of teriparatide (CinnoPar® 20 µg per day) along with calcium and vitamin D supplementation.

In the short-term follow-up, no complication was observed. Five weeks later, signs of the union were observed in the follow-up radiographs and the patient was finally able to bear weight for short periods of time on a daily bases. The patient allowed us to report her case and informed consent has been obtained in this regard.

## Discussion

Pregnancy-associated osteoporosis is a rarely observed skeletal pathology, which might lead to the fractures of femur before and after delivery (4). Femoral venous stasis due to pressure from the pregnant uterus, marrow hypertrophy, immobilization and the pressure of the pregnant uterus on the obturator nerve is the possible causes (2). Its potential complications can be prevented by limiting complete bed rest and prevention of weight-bearing activities. 

In this case report, we have described the diagnosis of postpartum atraumatic BFNF, in the context of excessive consumption of corticosteroid during pregnancy and subsequent osteoporosis. In addition to repeated courses of betamethasone injections, twin pregnancy, immobility and vitamin D deficiency could have contributed to the reduction of bone density in our patient. Besides orthopedic treatment, administration of teriparatide was effective in treating the patient. Bisphosphonates were used for treating transient osteoporosis of pregnancy in few case reports (5, 6), but the safety of bisphosphonates in premenopausal women with childbearing potential is unclear (7). So, we used teriparatide in the management of osteoporosis in our case. 

Taking into account the diagnosis of BFNF from other differentials of pelvic girdle pain in pregnant women is crucial, to prevent its complications. Furthermore, since hip fracture is reported during the labor of affected patients, cesarean section is preferred, provided the diagnosis is made before delivery (8). The incidence of pregnancy-associated osteoporosis is higher in twin pregnancies. So this complication should be kept in mind in these mothers (9).

Moreover, the decision for the administration of antenatal corticosteroids should be made carefully according to valid and current guidelines. As it might affect the quality of bone and could be considered as a possible cause of BFNF in our patient. Treating women with a history of a single miscarriage with oral prednisolone has not been shown to improve birth outcomes in literature. Moreover, the current guidelines recommend only single courses of steroid injections for fetal lung maturity in women at risk of preterm delivery (10).

## Conclusion

In conclusion, we suggest clinicians consider the pregnancy-associated osteoporosis and femoral neck fracture in the differential diagnosis of the painful hip during or after pregnancy, especially in twin pregnancies, in order to prevent further serious complications. Awareness of risk factors and avoiding the use of excessive and unnecessary corticosteroid might help to reduce the rate of BFNF.
